# Supplementary material for: Kinetic Study of CO2 Hydration by Small-Molecule Catalysts with A Second Coordination Sphere that Mimic the Effect of the Thr-199 Residue of Carbonic Anhydrase
Source: Biomimetics (Basel). 2019 Oct 1;4(4):66. doi: 10.3390/biomimetics4040066 (PMC6963681; doi:10.3390/biomimetics4040066)
Supplement: Supplementary file 1 [file biomimetics-04-00066-s001.pdf]

1. Materials synthesis

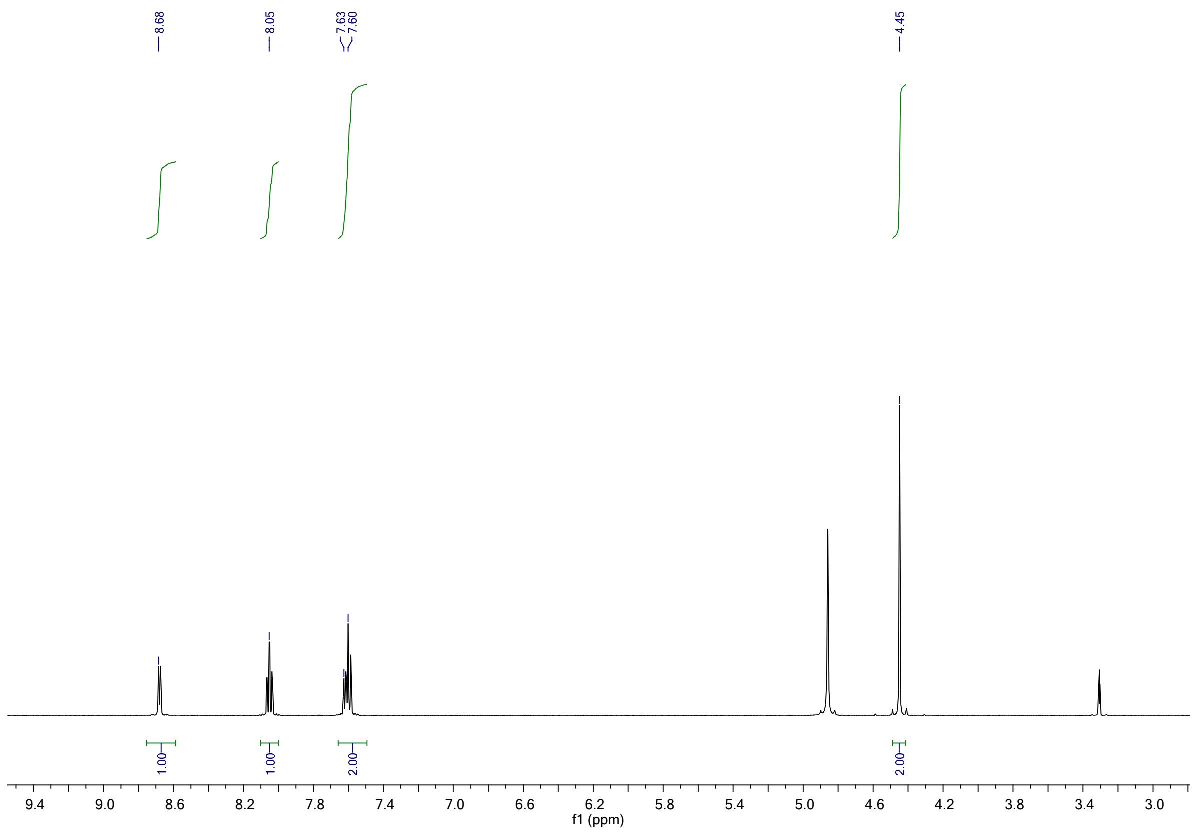

Figure S1. ^{1}H-NMR spectrum of $[(TPA)Zn(OH_2)](ClO_4)_2$ (**1**) in CD_3OD

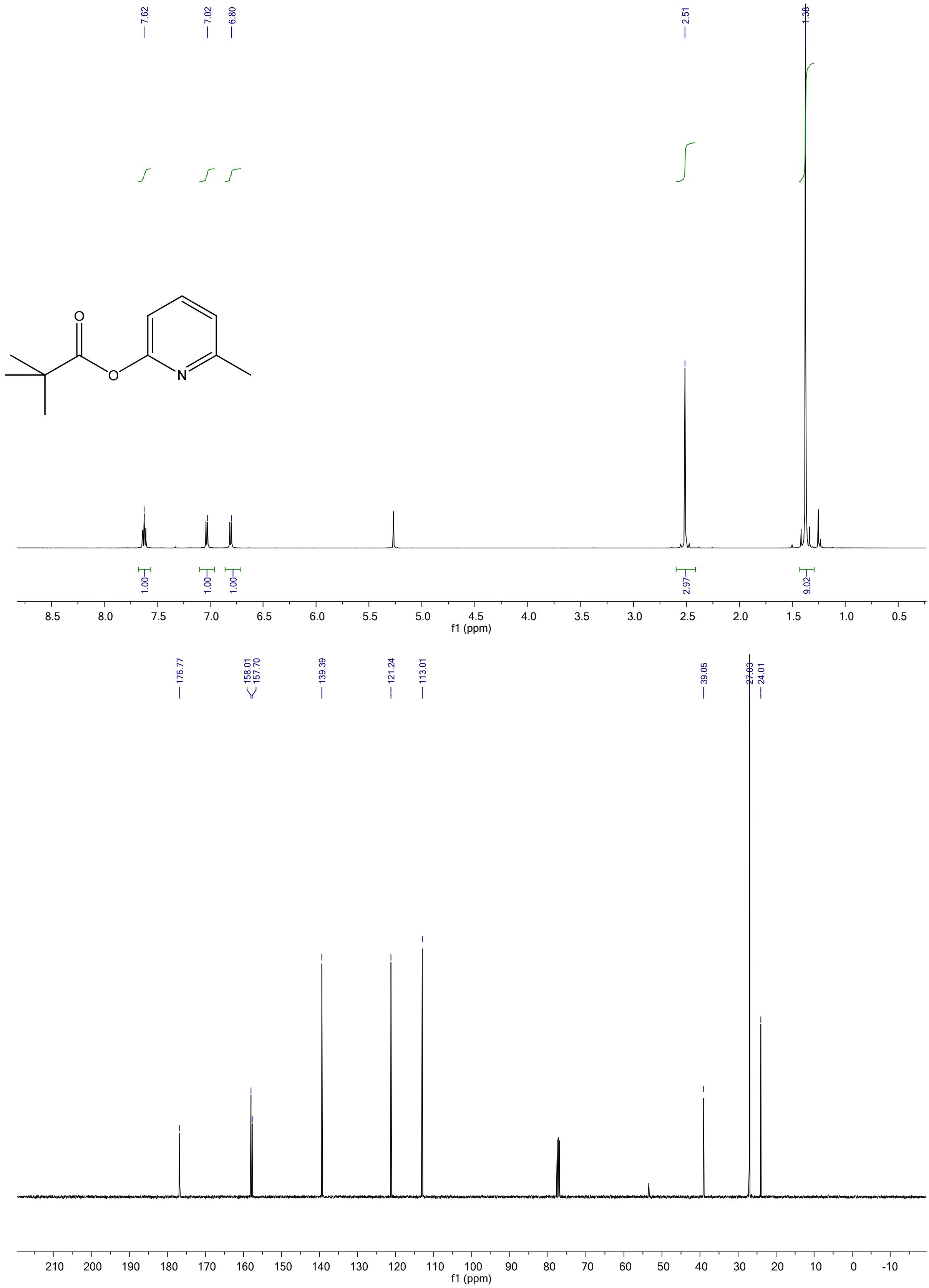

Figure S2. ^{1}H-NMR and ^{13}C-NMR spectra of 6-methylpyridine-2yl pivalate (**5**) in $CDCl_3$

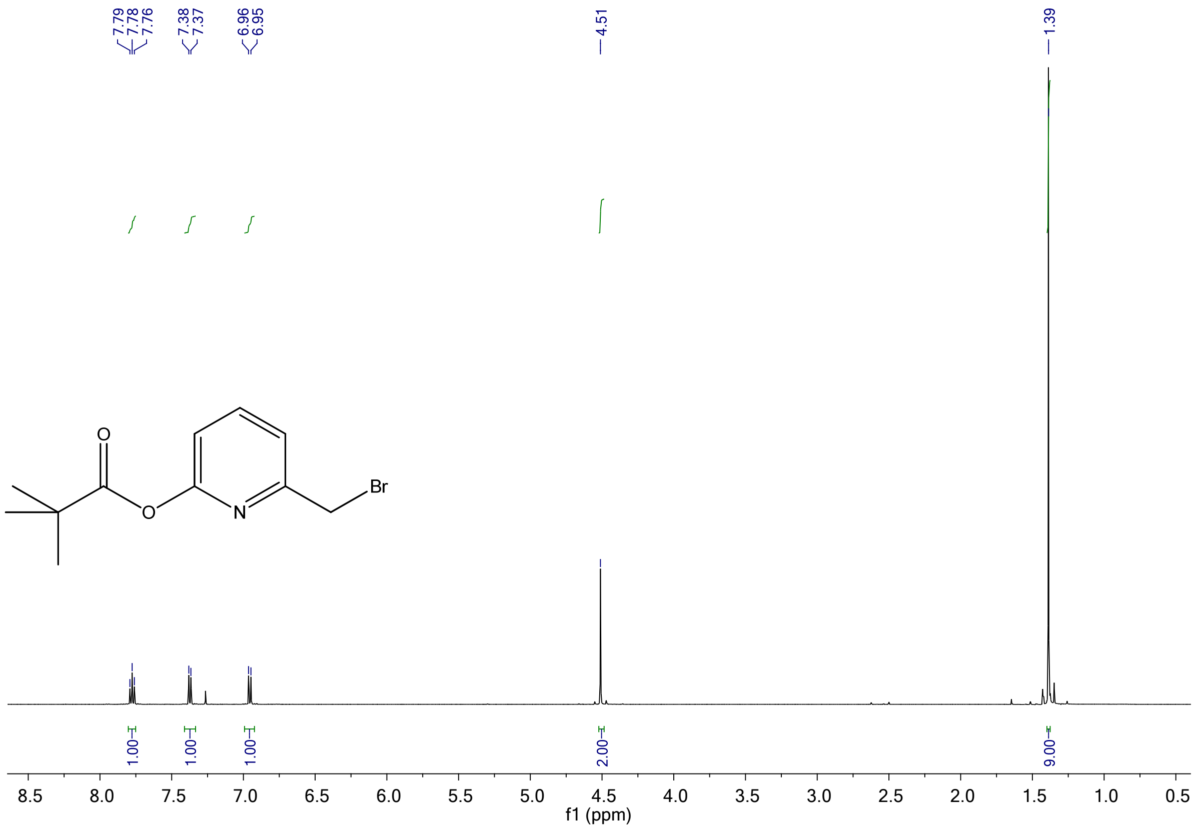

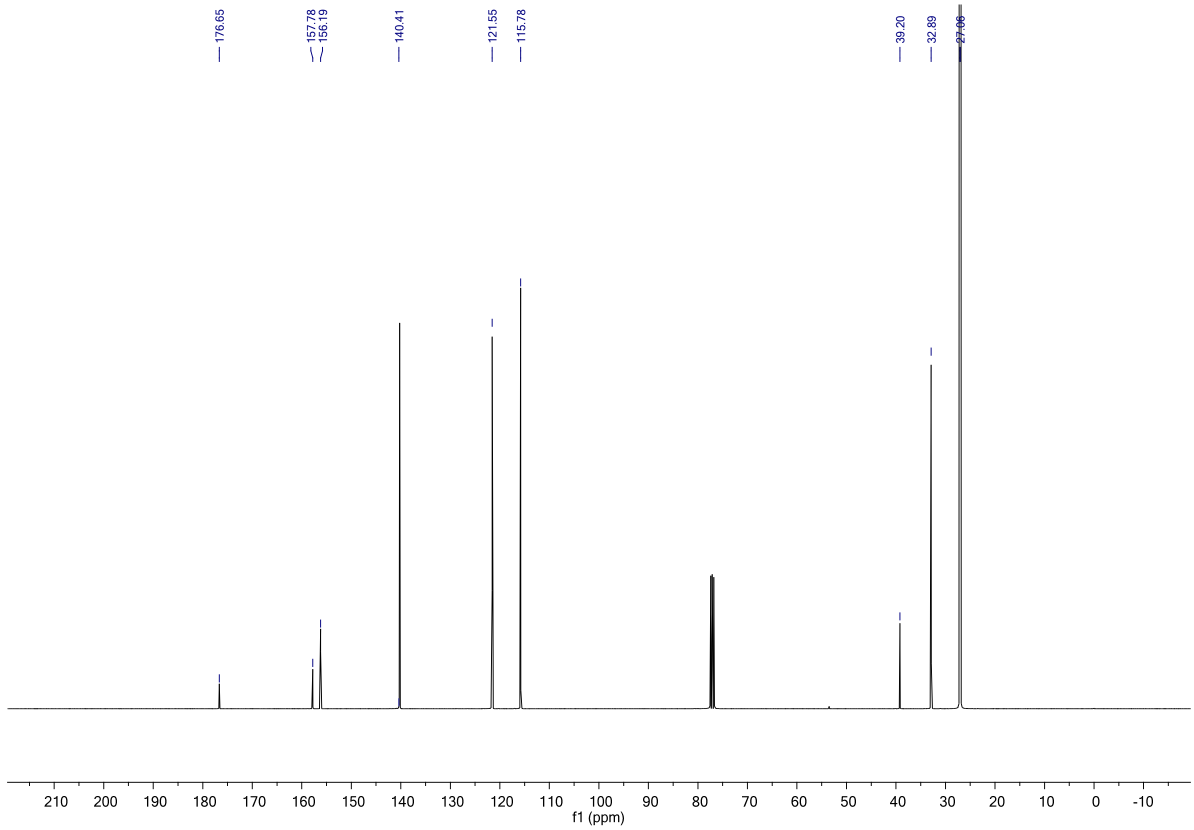

Figure S3. ^{1}H-NMR and ^{13}C-NMR spectra of 6-(bromomethyl)pyridine-2-yl pivalate (6) in $CDCl_3$

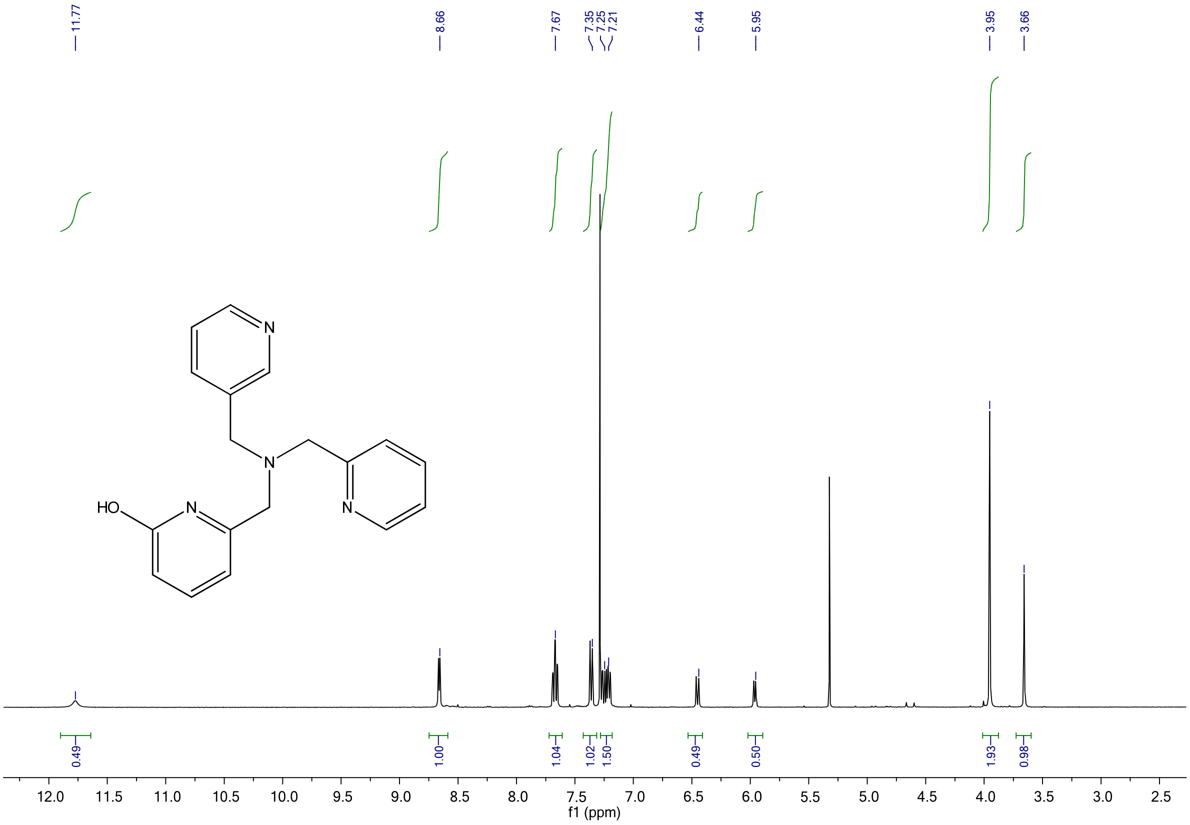

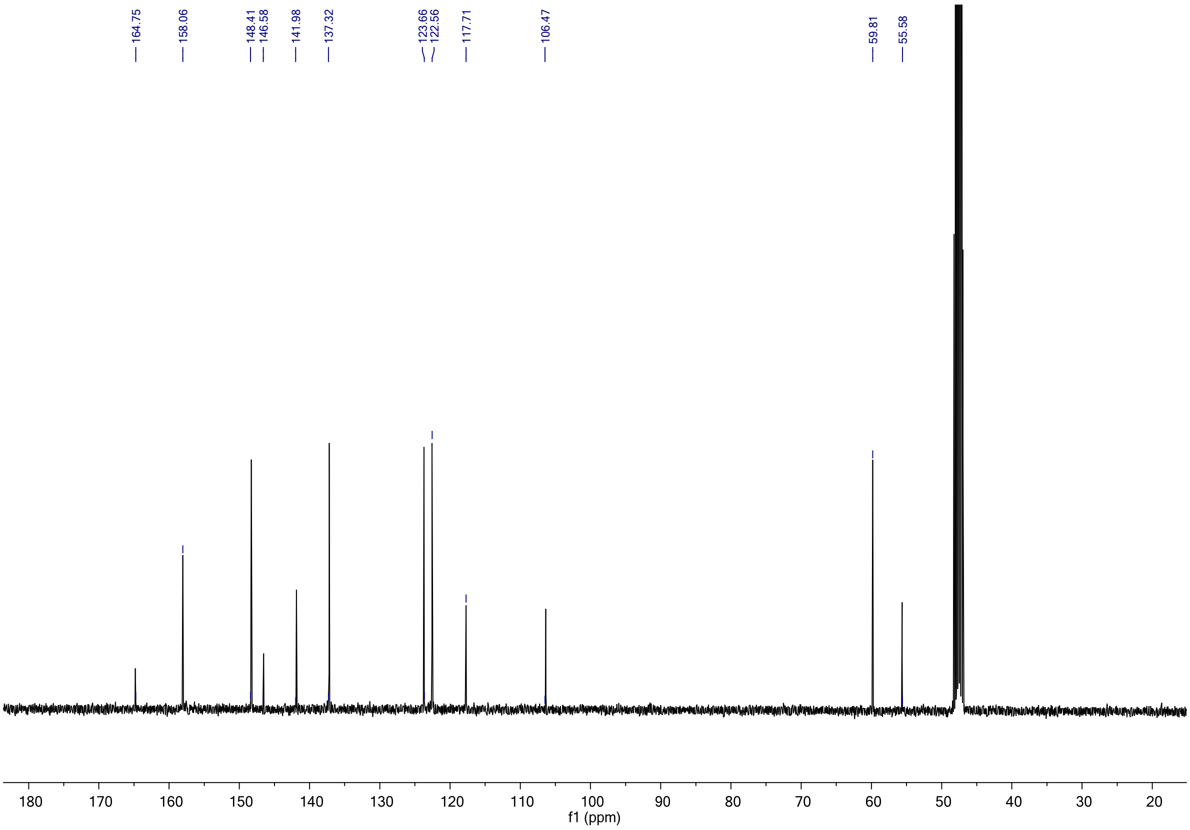

Figure S4. ^{1}H-NMR and ^{13}C-NMR spectra of 6-(((pyridin-2-ylmethyl)(pyridin-3-ylmethyl)amino)methyl)pyridin-2-ol (**9**) (TPA-OH) in CD_3OD

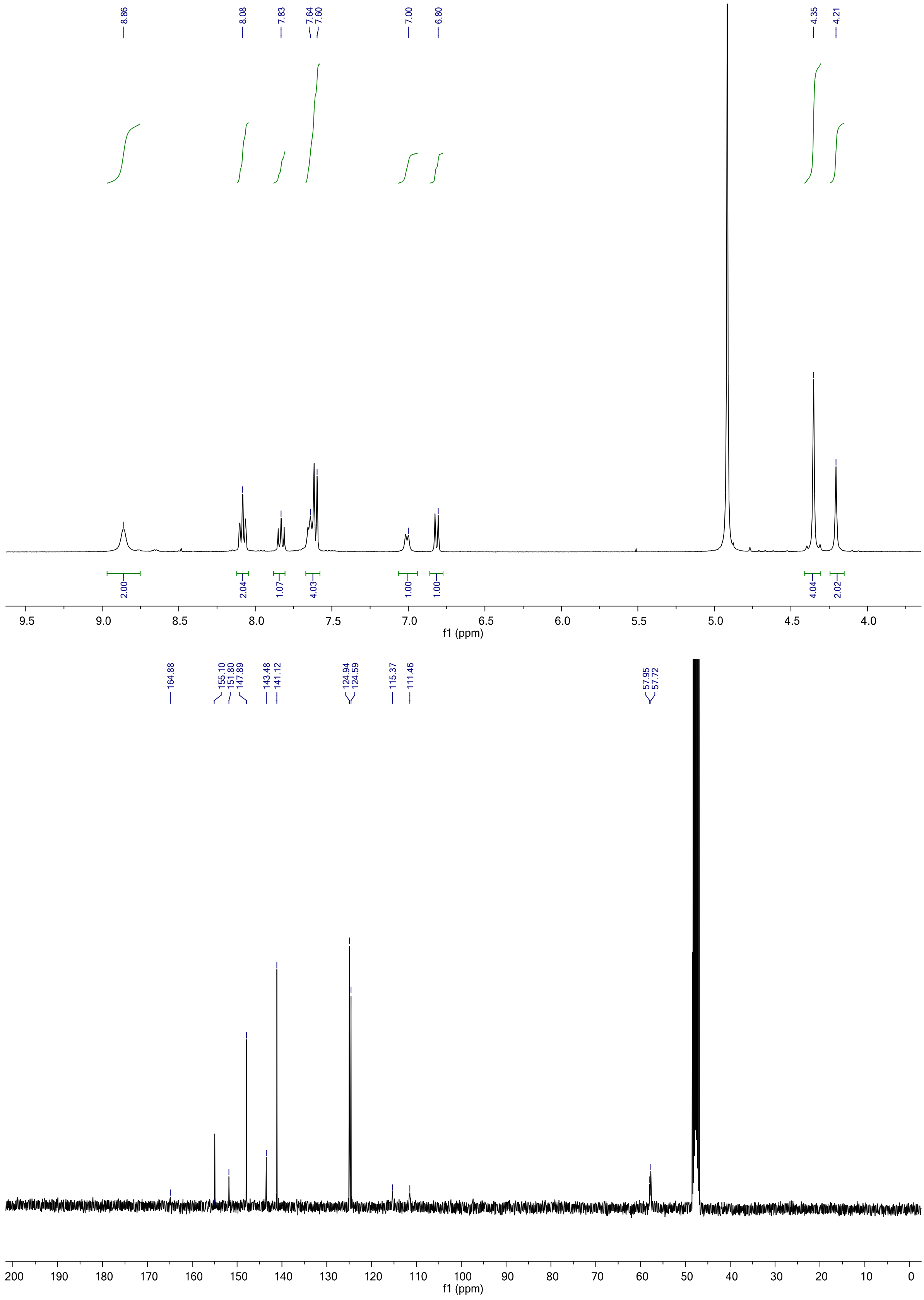

Figure S5. ^{1}H-NMR and ^{13}C-NMR spectra of [(TPA-OH)Zn(OH_2)](ClO_4)$_2$ (**2**) in CD_3OD

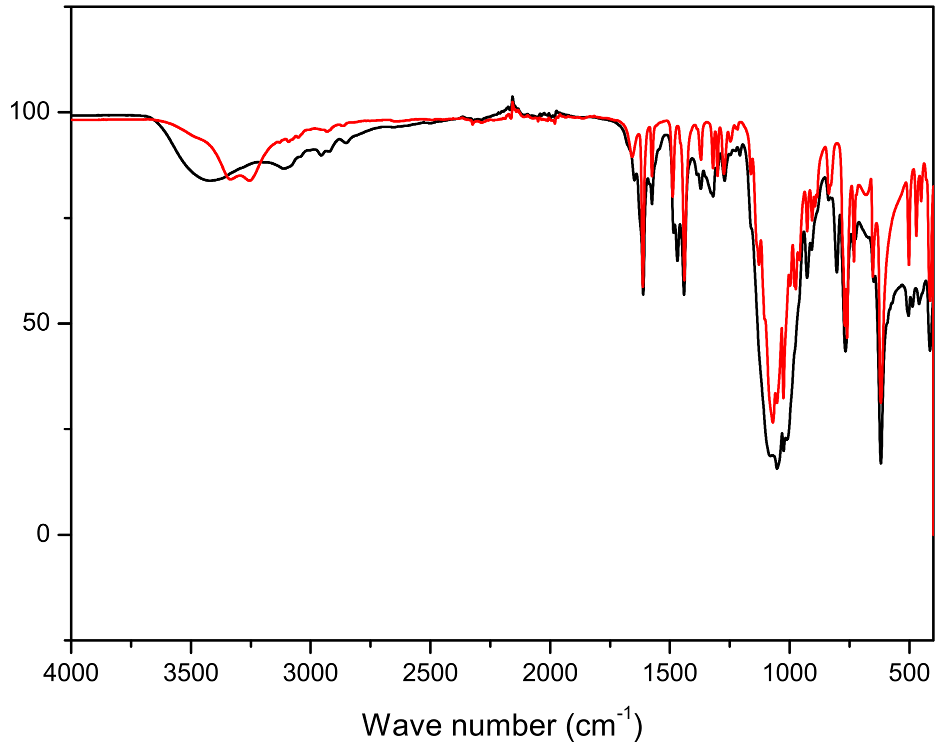

Figure S6. FT-IR spectra of (**1**) and (**2**). Red: (**1**), Black: (**2**)

2. Absorbance change of (1) and (2)

Trial	A_0	A_e	A_0-A_e	$d(\ln(A'-A_e))/dt$	-Q factor	V_{int}	k_{obs}
**(1)** - 1	0.55956	0.008081	0.551479	-0.35327	-0.0734	0.0142998604	1692.291171
**(1)** - 2	0.27742	0.010168	0.267252	-0.26744	-0.0734	0.005246182416	620.8499901
**(1)** - 3	0.27768	0.01109	0.26659	-0.29554	-0.0734	0.00578304	684.383412
**(1)** - 4	0.27449	0.011543	0.262947	-0.28587	-0.0734	0.00551738	652.9443269
**(1)** - 5	0.28415	0.012756	0.271394	-0.26954	-0.0734	0.005369323	635.4228337
**(2)** - 1	0.49676	0.037881	0.458879	-0.28831	-0.0734	0.009710776	1149.204295
**(2)** - 2	0.28235	0.030796	0.251554	-0.36684	-0.0734	0.006773357	801.5807208
**(2)** - 3	0.28303	0.03236	0.25067	-0.35064	-0.0734	0.006451488	763.4896774
**(2)** - 4	0.28202	0.03291	0.24911	-0.31199	-0.0734	0.005704635	675.1047859
**(2)** - 5	0.28121	0.032542	0.248668	-0.31593	-0.0734	0.005766427	682.4174441

3. Conversion between observed and pH-independent rates

Total concentration of catalyst equals

$[Zn_{tot}] = [Zn - OH^-] + [Zn - OH_2].$

The observed rate constant is given by,

$v_{init} = k_{obs}[Zn_{tot}][CO_2]$

where v_{init} is the initial velocity measured. The pH-independent rate constant is

$v_{init} = k_{ind}[Zn - OH^-][CO_2]$

where k_{ind} is the pH-independent form.

Combining the two equations yields,

$$k_{ind} = k_{obs}\frac{[Zn_{tot}]}{[Zn-OH^-]} = k_{obs}\left(\frac{[Zn-OH_2] + [Zn-OH^-]}{[Zn-OH^-]}\right) = k_{obs}\left(\frac{[Zn-OH_2]}{[Zn-OH^-]} + 1\right)$$

The fraction of deprotonated species is obtained using the Henderson–Hasselbalch equation.

$$pK_a - pH = \log\frac{[Zn - OH_2]}{[Zn - OH^-]}$$

Therefore, the relationship between k_{ind} and k_{obs} is given by,

$$k_{ind} = k_{obs}(10^{pK_a - pH} + 1)$$
